# 

**Published:** 1875-01

**Authors:** 


### CONTENTS:

OBITUARY:
James Van Zandt Blaney, A.M., M.D. 3
ORIGINAL COMMUNICATIONS:
ART. I. Medical Expert and Medical Evidence. By M. A. McClelland, M.D., Knoxville, Ill. .... 9
ART. II. Treatment of Dysentery with Creasote. By Jno. R. Cushing, M.D., Harrison Co., Texas .... 28
PROGRESS IN MEDICAL SCIENCES:
ART. I. Progress in Obstetrics and Gynæcology. By A. Reeves Jackson, M.D., Chicago .... 31
ART. II. Progress of Surgery. By Jno. E. Owens, M.D., Chicago .... 37
REPORTS OF SOCIETIES .... 44
HOSPITALS .... 48
CLINICS .... 54
EDITORS' BOOK TABLE .... 57
EDITORIAL .... 72
MORTALITY STATISTICS .... 78

